# *PPARG*, *KCNJ11*, *CDKAL1*, *CDKN2A-CDKN2B*, *IDE-KIF11-HHEX*, *IGF2BP2* and *SLC30A8* Are Associated with Type 2 Diabetes in a Chinese Population

**DOI:** 10.1371/journal.pone.0007643

**Published:** 2009-10-28

**Authors:** Cheng Hu, Rong Zhang, Congrong Wang, Jie Wang, Xiaojing Ma, Jingyi Lu, Wen Qin, Xuhong Hou, Chen Wang, Yuqian Bao, Kunsan Xiang, Weiping Jia

**Affiliations:** 1 Department of Endocrinology and Metabolism, Shanghai Jiao Tong University Affiliated Sixth People's Hospital, Shanghai, China; 2 Shanghai Diabetes Institute, Shanghai, China; 3 Shanghai Clinical Center for Diabetes, Shanghai, China; National Institute of Child Health and Human Development/National Institutes of Health, United States of America

## Abstract

**Background:**

Recent advance in genetic studies added the confirmed susceptible loci for type 2 diabetes to eighteen. In this study, we attempt to analyze the independent and joint effect of variants from these loci on type 2 diabetes and clinical phenotypes related to glucose metabolism.

**Methods/Principal Findings:**

Twenty-one single nucleotide polymorphisms (SNPs) from fourteen loci were successfully genotyped in 1,849 subjects with type 2 diabetes and 1,785 subjects with normal glucose regulation. We analyzed the allele and genotype distribution between the cases and controls of these SNPs as well as the joint effects of the susceptible loci on type 2 diabetes risk. The associations between SNPs and type 2 diabetes were examined by logistic regression. The associations between SNPs and quantitative traits were examined by linear regression. The discriminative accuracy of the prediction models was assessed by area under the receiver operating characteristic curves. We confirmed the effects of SNPs from *PPARG*, *KCNJ11*, *CDKAL1*, *CDKN2A-CDKN2B*, *IDE-KIF11-HHEX*, *IGF2BP2* and *SLC30A8* on risk for type 2 diabetes, with odds ratios ranging from 1.114 to 1.406 (*P* value range from 0.0335 to 1.37E-12). But no significant association was detected between SNPs from *WFS1, FTO, JAZF1*, *TSPAN8-LGR5*, *THADA*, *ADAMTS9*, *NOTCH2-ADAM30* and type 2 diabetes. Analyses on the quantitative traits in the control subjects showed that *THADA* SNP rs7578597 was association with 2-h insulin during oral glucose tolerance tests (*P* = 0.0005, empirical *P* = 0.0090). The joint effect analysis of SNPs from eleven loci showed the individual carrying more risk alleles had a significantly higher risk for type 2 diabetes. And the type 2 diabetes patients with more risk allele tended to have earlier diagnostic ages (*P* = 0.0006).

**Conclusions/Significance:**

The current study confirmed the association between *PPARG*, *KCNJ11*, *CDKAL1*, *CDKN2A-CDKN2B*, *IDE-KIF11-HHEX*, *IGF2BP2* and *SLC30A8* and type 2 diabetes. These type 2 diabetes risk loci contributed to the disease additively.

## Introduction

Type 2 diabetes is a complex disease characterized by elevated blood glucose, caused mainly by impairment in both insulin action and beta cell function. Although the sharp increase in prevalence of type 2 diabetes worldwide is attributed to changes in individual environmental exposure pattern, genetic factors do predispose to the disease [1]. Recently, spectacular advance was made in identifying susceptible genes involved in type 2 diabetes through genome-wide association studies (GWAS). Several groups reported independent GWAS in Caucasians, which not only confirmed the effect of *PPARG*, *KCNJ11* and *TCF7L2*, but also identified six novel susceptibility loci including *CDKAL1*, *CDKN2A-CDKN2B*, *IDE-KIF11-HHEX*, *IGF2BP2*, *SLC30A8* and *FTO* [2], [3], [4], [5]. And meta-analysis on three Caucasian GWAS yielded six additional loci (*JAZF1*, *TSPAN8-LGR5*, *THADA*, *ADAMTS9*, *NOTCH2-ADAM30* and *CDC123-CAMK1D*) associated with type 2 diabetes at genome-wide statistic significance [6]. Moreover, the first two independent GWAS in East Asian population added *KCNQ1* to the list of type 2 diabetes susceptible gene [7], [8]. In addition with two well replicated candidate genes *TCF2* and *WFS1* [9], [10], eighteen susceptible loci of type 2 diabetes were well recognized to date.

Several researches tried to replicate some of these loci in the Asian populations and confirmed the effects of genetic variants in *CDKAL1*, *CDKN2A-CDKN2B*, *IDE-KIF-HHEX*, *IGF2BP2*, *SLC30A8*, *FTO* and *TCF7L2* [11], [12], [13]. However, limited study analyzed all these susceptible loci in the Asian population. Previously, we reported the association of *TCF2* and *KCNQ1* genetic variants with type 2 diabetes in our case-control samples [14], [15], thus we tested the effects of genetic variants from the other sixteen loci as well as the joint effect of variants from all these eighteen loci on type 2 diabetes and related clinical traits in this study.

## Methods

### Ethnic statement

This study was approved by the institutional review board of Shanghai Jiao Tong University Affiliated Sixth People's Hospital was in accordance with the principle of the Helsinki Declaration II. Written informed consent was obtained from each participant.

### Study population

A total of 3,634 individuals, comprising 1,849 type 2 diabetes patients and 1,785 normal controls, were included in the present study. The type 2 diabetes patients were recruited from the inpatient department of Shanghai Jiao Tong University Affiliated Sixth People's Hospital. Diabetes was defined according to 1999 World Health Organization criteria (fasting plasma glucose ≥7.0 mmol/l and/or 2-h plasma glucose ≥11.1 mmol/l) [16]. Individuals with glutamic acid decarboxylase and/or protein tyrosine phosphatase IA-2 antibodies positive were excluded and mitochondria tRNA ^Leu(UUR)^ nt3243 A-to-G mutation carriers were excluded as well. The controls were selected from the participants of Shanghai Diabetes Study [17]. In the present study, the inclusion criteria for the control subjects were: 1) over 40 years old; 2) with normal glucose regulation confirmed by a standard 75 g oral glucose tolerance test (OGTT); 3) with negative family history of diabetes by a standard questionnaire. For each participant, glucose tolerance status was confirmed according to 1999 World Health Organization criteria (fasting plasma glucose <6.1 mmol/l and 2-h postprandial plasma glucose <7.8 mmol/l). The clinical characteristics of the study groups were shown in Table 1.

**Table 1:** Clinical characteristics of study population.

	Type 2 diabetes patients	Normal glucose regulation subjects
Samples (*n*)	1,849	1,785
Male/Female (*n*)	970/879	736/1,049
Age (years)	61.21±12.62	57.39±12.37
Duration of diabetes (years)	6.0 (1.0, 10.0)	
BMI (kg/m^2^)	24.04±3.51	23.57±3.25

Data are shown as mean±SD or median (interquartile range).

At the level of significance of 0.05, our case-control samples had over 80% power to detect a minimum odds ratio (OR) of 1.15 for a single nucleotide polymorphism (SNP) with minor allele frequency (MAF) over 0.2, a minimum OR of 1.24 for a SNP with MAF of 0.1 and a minimum OR of 1.33 for a SNP with MAF of 0.05.

### Biochemical and anthropometric measures

All participants underwent a detailed clinical investigation. Anthropometric variables including height, weight, waist and hip circumferences were measured. For the controls, blood samples were obtained at 0 and 120 min during standard 75 g OGTTs. Plasma glucose concentrations were measured by the glucose oxidase-peroxidase method using commercial kits (Shanghai Biological Products Institution, Shanghai, China). Serum insulin levels were measured by radioimmunoassay (Linco Research, St Charles, MO, USA). Homeostasis model assessment (HOMA) was used to estimate insulin resistance (HOMA-IR) and β-cell function (HOMA-B) [18].

### SNP selection and genotyping

We selected twenty-four SNPs from sixteen loci previously reported to be associated with type 2 diabetes at a genome-wide significance level, including *PPARG* (rs1801282), *KCNJ11* (rs5219), *WFS1* (rs10010131), *TCF7L2* (rs7903146), *CDKAL1* (rs10946398, rs7754840, rs9460546, rs7756992 and rs9465871), *CDKN2A-CDKN2B* (rs564398 and rs10811161), *IDE-KIF11-HHEX* (rs10509645, rs1111875 and rs10748582), *IGF2BP2* (rs7651090), *SLC30A8* (rs13266634), *FTO* (rs8050136), *JAZF1* (rs864754), *TSPAN8-LGR5* (rs7961581), *THADA* (rs7578597), *ADAMTS9* (rs4607103), *NOTCH2-ADAM30* (rs2641348 and rs10923931) and *CDC123-CAMK1D* (rs12779790). All the SNPs were genotyped using MassARRAY iPLEX system (MassARRAY Compact Analyzer, Sequenom, San Diego, CA). The genotyping underwent several quality control procedures. Two SNPs (rs12779790 and rs7903146) were excluded because of low call rate (<90%). One SNP (rs10923931) was excluded because of departure from Hardy-Weinberg equilibrium (*P*<0.01). The overall call rate for the remaining 21 SNPs was 96.8%. The concordant rate calculated based on 100 duplicates for each SNP was 99.6%.

### Statistical analyses

Allele frequencies between cases and controls were compared using χ^2^ test. The ORs with 95% confidence intervals (CIs) with respect to the risk alleles were presented. Genotype distributions between cases and controls were compared using logistic regression under a log additive model in PLINK after adjusting age, gender and BMI as confounding factors [19]. For genes with multiple SNPs genotyped, independent SNP effects were determined by logistic regression. Correction of multiple testing on allele association was performed using PLINK through 10,000 permutations that randomly permutated the case/control status independent of genotypes.

For continuous trait analysis, quantitative trait association analyses were performed by linear regression after adjusting age, gender and BMI as confounding factors. Quantitative traits with skewed distribution were transformed to approximate univariate normality by natural logarithm. In order to adjust multiple comparison, 1,000 permutations were performed for each trait to assess empirical *P* values using PLINK [19]. Additive effect models were used for SNPs with MAFs >0.05. For SNPs with MAFs <0.05, dominant effect models were used because of the small numbers of homozygotes of rare alleles.

The receiver operating characteristic (ROC) curves, a tool for displaying the discriminatory ability of a diagnostic marker in distinguishing between cases and controls, were used for evaluation of discriminative accuracy of age, gender, BMI and SNPs. The area under the ROC curve (AUC), the measurement of the ability of a marker to discriminate between the cases and controls, was calculated by using the logistic regression model. AUC comparisons between ROC curves were performed by MedCalc (version 10.3.2.0; Mariakerke, Belgium) using the method of Hanley and McNeil for ROC curve analyses.

The statistic analyses were performed by using SAS for Windows (version 8.0; SAS Institute, Cary, NC) unless specified otherwise. As the genes analyzed in the current study were well replicated previously, a two-tailed *P* value of <0.05 was considered as statistically significant.

## Results

We first examined the potential effects of 21 SNPs successfully genotyped on type 2 diabetes susceptibility in our Chinese case-control samples. As shown in Table 2, the *CDKN2A-CDKN2B* rs10811661 showed strong association to type 2 diabetes, with an OR of 1.406 (95% CI 1.280–1.546) per risk allele T (*P* = 1.37E-12). In addition, SNPs from *PPARG*, *KCNJ11*, *CDKAL1*, *IDE-KIF11-HHEX*, *IGF2BP2* and *SLC30A8* were also moderately associated with type 2 diabetes, with ORs ranging from 1.114 to 1.282 (*P*<0.05). As multiple SNPs from *CDKAL1* and *IDE-KIF11-HHEX* were genotyped, we analyzed the linkage disequilibrium extent among the SNPs from the same region. The five SNPs from *CDKAL1* and three SNPs from *IDE-KIF11-HHEX* were in linkage disequilibrium, respectively. We then used logistic regression to determine the independent effects of these SNPs and found SNPs rs7756992 and rs10748582 conferred independent risks for the disease in *CDKAL1* and *IDE-KIF11-HHEX* regions respectively (data not shown). However, after correction of multiple comparisons, only the association between SNPs from *CDKAL1*, *CDKN2A-CDKN2B*, *IDE-KIF11-HHEX*, *IGF2BP2* and *SLC30A8* and type 2 diabetes remained to be significant. And we also found the *WFS1* rs10010131 and *FTO* rs8050136 showed trends towards association to type 2 diabetes in our samples (0.05<*P*<0.1). However, we failed to replicate the effects of *CDKN2A-CDKN2B* rs564398 and SNPs from *JAZF1*, *TSPAN8-LGR5*, *THADA*, *ADAMTS9*, *NOTCH2-ADAM30*, which were identified through meta-analysis of Caucasian GWAS.

**Table 2:** Association between SNPs from fourteen loci and type 2 diabetes in the Chinese.

Gene	SNP	Chromosome	Chromosome position (Build 36)	Major/Minor Allele	Risk Allele	Risk allele frequency		OR (95%CI)	*P*_allele_	*P*_genotype_	Empirical *P*
						Case	Control				
*PPARG*	rs1801282	3	12368125	C∶G	C	0.950	0.939	1.246 (1.017–1.526)	0.0335	0.0701	0.4589
*KCNJ11*	rs5219	11	17366148	C∶T	T	0.425	0.394	1.138 (1.034–1.251)	0.0079	0.0031	0.1367
*WFS1*	rs10010131	4	6343816	G∶A	G	0.955	0.946	1.213 (0.975–1.510)	0.0824	0.0969	0.7816
*CDKAL1*	rs10946398	6	20769013	A∶C	C	0.441	0.414	1.114 (1.014–1.224)	0.0241	0.0074	0.3551
*CDKAL1*	rs7754840	6	20769229	G∶C	C	0.440	0.411	1.127 (1.027–1.238)	0.0119	0.0025	0.1967
*CDKAL1*	rs9460546	6	20771611	T∶G	G	0.444	0.411	1.145 (1.041–1.260)	0.0054	0.0014	0.0944
*CDKAL1*	rs7756992	6	20787688	G∶A	G	0.548	0.511	1.158 (1.056–1.272)	0.0020	0.0010	0.0364
*CDKAL1*	rs9465871	6	20825234	C∶T	C	0.554	0.522	1.140 (1.039–1.251)	0.0057	0.0021	0.1001
*CDKN2A-CDKN2B*	rs564398	9	22019547	A∶G	G	0.120	0.118	1.020 (0.885–1.177)	0.7826	0.2922	1
*CDKN2A-CDKN2B*	rs10811161	9	19269853	T∶C	T	0.604	0.520	1.406 (1.280–1.546)	1.37E-12	1.13E-13	0.0001
*IDE-KIF11-HHEX*	rs10509645	10	94267846	A∶C	C	0.354	0.321	1.160 (1.052–1.280)	0.0031	0.0018	0.0561
*IDE-KIF11-HHEX*	rs1111875	10	94452862	A∶G	G	0.310	0.273	1.201 (1.085–1.330)	0.0004	5.25E-05	0.0086
*IDE-KIF11-HHEX*	rs10748582	10	94467199	A∶T	T	0.238	0.196	1.282 (1.146–1.435)	1.46E-5	1.51E-05	0.0005
*IGF2BP2*	rs7651090	3	186996086	A∶G	G	0.282	0.246	1.200 (1.079–1.334)	0.0008	0.0013	0.0141
*SLC30A8*	rs13266634	8	118253964	C∶T	C	0.613	0.559	1.251 (1.138–1.374)	3.12E-6	1.60E-06	0.0002
*FTO*	rs8050136	16	52373776	C∶A	A	0.130	0.118	1.125 (0.978–1.294)	0.0996	0.1456	0.8414
*JAZF1*	rs864754	7	25918763	T∶A	T	0.759	0.751	1.046 (0.934–1.171)	0.4364	0.4359	0.9998
*TSPAN8-LGR5*	rs7961581	12	69949369	T∶C	C	0.231	0.217	1.082 (0.965–1.212)	0.1779	0.4068	0.9710
*THADA*	rs7578597	2	43586327	T∶C	T	0.994	0.994	1.013 (0.548–1.873)	0.9661	0.7604	1
*ADAMTS9*	rs4607103	3	64686944	C∶T	C	0.635	0.629	1.030 (0.934–1.137)	0.5510	0.8632	1
*NOTCH2-ADAM30*	rs2641348	1	120239407	T∶C	C	0.028	0.026	1.071 (0.801–1.433)	0.6443	0.9776	1

The OR with 95% CI shown is for the risk allele. *P*_allele_ is the *P* value for comparison of the allele distribution between the cases and controls. *P*_genotype_ is the *P* value for comparison of genotype distribution between the cases and controls after adjusting age, gender and BMI as confounding factors. Empirical *P* values were calculated through 10,000 permutations for the difference of allele distribution between cases and controls.

We then analyzed the effects of these SNPs on clinical measurements in the normal controls. As shown in Table 3, we found *SLC30A8* rs13266634 was associated with fasting glucose (*P* = 0.0118) and *TSPAN8-LGR5* rs7961581 was associated with 2-h glucose (*P* = 0.0404). For insulin levels, we found rs10811161, rs864754, rs7961581 and rs7578597 were associated with 2-h insulin levels (*P* = 0.0005∼0.0257). For BMI, no significant association was detected, including *FTO* SNP rs8050136, which was previously reported to be an obesity gene in the Caucasians. However, only the association between *THADA* rs7578597 and 2-h insulin remained significant after correction of multiple testing (empirical *P* = 0.0090).

**Table 3:** Association of SNPs with clinical features related to glucose metabolism in the control subjects.

Traits	Gene	SNP	AA	Aa	aa	*P*	Empirical *P*
Fasting glucose (mmol/l)	*SLC30A8*	rs13266634	5.02±0.02	4.99±0.02	4.93±0.03	0.0118	0.1698
2-h glucose (mmol/l)	*TSPAN8-LGR5*	rs7961581	5.42±0.04	5.48±0.05	5.75±0.14	0.0404	0.3546
Fasting insulin (pmol/l)	*TSPAN8-LGR5*	rs7961581	45.31±0.98	52.31±1.79	47.80±4.16	0.0054	0.0759
2-h insulin (pmol/l)	*CDKN2A-CDKN2B*	rs10811161	241.69±8.74	257.93±7.21	284.54±14.22	0.0257	0.2927
2-h insulin (pmol/l)	*JAZF1*	rs864754	268.96±7.11	248.23±8.35	225.81±15.18	0.0110	0.1489
2-h insulin (pmol/l)	*TSPAN8-LGR5*	rs7961581	247.86±6.16	268.68±8.80	323.46±53.78	0.0171	0.2088
2-h insulin (pmol/l)	*THADA*	rs7578597	258.90±5.05	165.79±39.03	/	0.0005	0.0090
HOMA-IR	*TSPAN8-LGR5*	rs7961581	1.66±0.04	1.93±0.07	1.75±0.16	0.0039	0.0609

Only SNPs showed nominal significant association to clinical features are shown in the table. AA represents the homozygotes of major allele. Aa represents the heterozygotes. aa represents the homozygotes of minor allele. Empirical *P* values were calculated through 1,000 permutations within each trait.

To further investigate if these susceptible loci affected the disease additively, we examined joint effects of risk alleles of SNPs from susceptible loci on type 2 diabetes. Here we analyzed if the individuals carrying more risk allele tended to have a higher risk for the disease and if the patients with more risk alleles tended to have an earlier diagnostic age. We also analyzed the diagnostic value of these SNPs. We only selected SNPs with *P* values less than 0.1. For loci with multiple SNPs genotyped, the SNP showed strongest association from each locus was selected. Two additional SNPs, *KCNQ1* rs2237892 and *TCF2* rs4430796, which were previously genotyped and showed significant association to type 2 diabetes in our samples, were also included into the combined analysis. Totally eleven SNPs (*PPARG* rs1801282, *KCNJ11* rs5219, *WFS1* rs10010131, *CDKAL1* rs7756992, *CDKN2A-CDKN2B* rs10811161, *IDE-KIF11-HHEX* rs10748582, *IGF2BP2* rs7651090, *SLC30A8* rs13266634, *FTO* rs8050136, *KCNQ1* rs2237892 and *TCF2* rs4430796) were selected into the analysis. And we only included individuals with the genotypes of all these eleven SNPs available (1,359 type 2 diabetes patients and 1,532 normoglycaemic controls) into the analysis.

**Figure 1:**
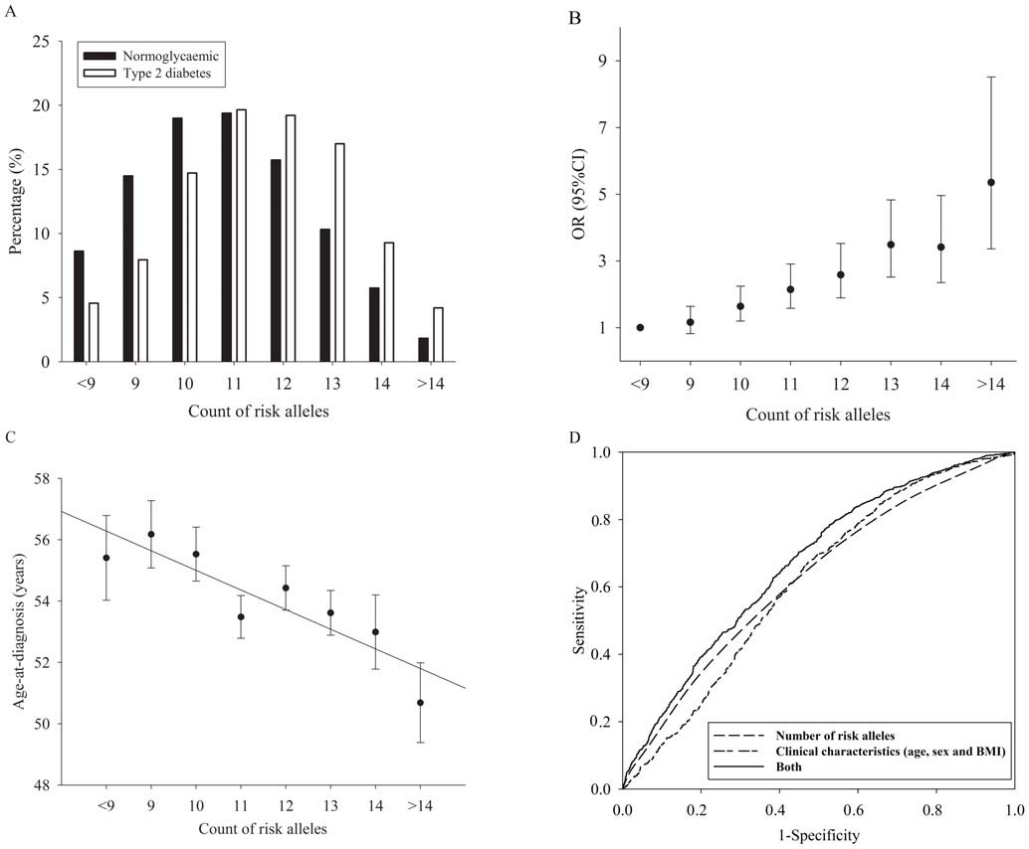
The joint effect of risk alleles on type 2 diabetes susceptibility. A. Distribution of number of risk alleles in the type 2 diabetes patients and normoglycaemic controls. Black bar = normoglycaemic, white bar = type 2 diabetes. The proportion of type 2 diabetes patients increased in the subgroups with more risk alleles (*P*_trend_ = 1.34E-30). B. The risk for type 2 diabetes increased according to the increase of number of risk alleles, (OR 1.265 per each additional risk allele, *P* = 2.66E-29). C. The age-at-diagnosis decreased according to the increase of number of risk alleles carried (−0.60±0.17 years per each additional risk allele, *P* = 0.0006). D. ROC curves for models containing number of risk alleles (AUC = 0.621), clinical characteristics including age, sex and BMI (AUC = 0.614) and both (AUC = 0.668).

We found the proportion of type 2 diabetes patients increased in the subgroups with more risk alleles (Fig. 1A) (*P*_trend_ = 1.34E-30). When treating the individuals carrying less than 9 risk alleles (6.6% of the study population) as reference group, the subgroups carrying more risk alleles had a significantly higher risk for type 2 diabetes, with each additional risk allele increased type 2 diabetes risk by 1.265-fold (95%CI: 1.214–1.318, *P* = 2.66E-29). Moreover, we found the number of risk alleles was significantly associated with age-at-diagnosis in the type 2 diabetes patients. The patients carrying more risk alleles tended to have a younger diagnostic age (β = −0.60±0.17 years per each additional risk allele, *P* = 0.0006) (Fig. 1C). And this association remained significant after further adjusting sex and BMI as confounding factors (β = –0.65±0.18 years per risk allele, *P* = 0.0002). We also evaluated the predictive value of these genetic variants in the Chinese population. In our samples, the AUC for clinical characteristic including age, sex and BMI was 0.614 (95%CI 0.595–0.632) while the AUC for the number of risk alleles was 0.621 (95%CI 0.604–0.639). But when adding the number of risk alleles to the regression model of age, sex and BMI, the AUC increased marginally to 0.668 (95%CI 0.650–0.685) (*P* = 0.0002) (Fig. 1D). We also analyzed the joint effect of SNPs from all sixteen loci (only excluding *TCF7L2* and *CDC123-CAMK1D* that failed genotyping) and got similar results (data not shown).

## Discussion

In this study, we confirmed the effects of variations in *PPARG*, *KCNJ11*, *CDKAL1*, *CDKN2A-CDKN2B*, *IDE-KIF11-HHEX*, *IGF2BP2* and *SLC30A8* on type 2 diabetes. The ORs of the risk alleles were similar to those reported in Caucasians [2], [3], [4], [5]. Our finding also supported that *WFS1* and *FTO* might participate in the pathogenesis of type 2 diabetes, although the effects we detected on rs10010131 and rs8050136 were not statistically significant. The non-significance could be explained by the different genetic background between Caucasian and Asian populations. Compared with Caucasians, the MAFs of these two variants were much rarer in the Chinese populations (0.05 vs. 0.40 for rs10010131 and 0.125 vs. 0.45 for rs8050136) [20], [21], which reduced the statistical power of our samples dramatically. It should also be noted that the non-significance of *FTO* SNP may be partly explained by the similar BMI between the cases and controls in our samples, as *FTO* variant increases type 2 diabetes risk mainly through increasing BMI. However, we failed to replicate the effects of SNPs reported by DIAGRAM study. As these loci were identified by a meta-analysis in over 10,000 individuals and replicated in over 50,000 independent samples [6], the effect sizes of these genes were relatively low compared with other loci. Moreover, several SNPs (rs7578597spor and rs2641348) were rare variants in the Asian populations. Therefore, we did not have enough power to replicate these associations in our samples. But the risk alleles of all the SNPs were the same as reported in the Caucasians [6].

We also found rs7578597, a non-synonymous variant of *THADA*, was associated with 2-h insulin levels in the controls. Evidence showed *THADA* might be involved in the death receptor pathway and apoptosis [22]. But whether *THADA* has similar effect on beta cell and how it participates in the pathogenesis of type 2 diabetes and insulin secretion is still unknown. Several study suggested the predominant effect of the genetic contribution to type 2 diabetes was mediated through defect in insulin secretion rather than action [23], [24]. But none of the SNPs we analyzed showed association to HOMA-B. Wu et al [12] reported that SNPs from *CDKN2A-CDKN2B*, *IGF2BP2* and *SLC30A8* showed association to beta cell function in the Chinese; however, negative finding was also reported in the current study and another study with equivalent sample size [11]. Thus the effects of these variants on clinical characteristics in the Chinese still need to be investigated in cohorts with more samples.

The joint effect analysis showed that these susceptible loci worked in an additive way and the subgroup of populations with more risk alleles tended to have a higher risk for the disease. However, the predictive value of these risk variants was limited in our samples. This was similar to the previous findings in Caucasians [25], [26]. But we found the patients with more risk alleles tended to have an earlier onset age of diabetes. Whether individuals with extremely more risk alleles could benefit from genetic testing and whether lifestyle intervention could reduce the risk in this subgroup of individuals remained to be investigated.

Although the findings presented are interesting, they should be viewed with caution. First, we cannot fully exclude the possibility the population stratification exists in our samples. Although both the cases and controls were recruited from the same city, recent large-scale migration from other regions of the country into Shanghai may lead to some degree of stratification. However, according to the records of our standard questionnaires, all of our samples lived in Eastern China more than three generations, thus the effect of population stratification may be limited in our samples. Second, as multiple traits were analyzed in the current study, the possibility still exists that our findings were false positive, especially for the continuous traits which lack replication in other independent samples.

In conclusion, we confirmed the association between *PPARG*, *KCNJ11*, *CDKAL1*, *CDKN2A-CDKN2B*, *IDE-KIF11-HHEX*, *IGF2BP2*, *SLC30A8* variants and type 2 diabetes. These risk loci contributed to type 2 diabetes additively. Further studies in Asian cohorts with prospective data and information on environmental factors are needed to better elucidate the effects of these genetic variants on diabetes risk and their interaction with environment.
